# Power ultrasound-driven silane-modified cement paste: Insights into microstructural evolution, hydrophobic and mechanical performance

**DOI:** 10.1016/j.ultsonch.2025.107591

**Published:** 2025-09-28

**Authors:** Guangqi Xiong, Ying Zhao, Zheng Fang, Yi Li, Yuanliang Ren, Xiaolong Jia, Bo Ran, Lei Xu, Shuai Zhou, Chong Wang

**Affiliations:** a College of Materials Science and Engineering, Chongqing University, Chongqing 400045, China; b Department of Civil and Environmental Engineering & Research Centre for Resources Engineering towards Carbon Neutrality, The Hong Kong Polytechnic University, Hong Kong 999077, China; c Laboratory of Construction Materials, Ecole Polytechnique Fédérale de Lausanne, 1015, Switzerland; d Institute of Industrial Science, The University of Tokyo, Komaba 4-6-1, Meguro-ku, Tokyo 153-8505, Japan; e College of Materials Science and Engineering, Southeast University, Nanjing 211189, China; f State Key Laboratory of Hydroscience and Engineering, Tsinghua University, Beijing 100084, China

**Keywords:** Ultrasonic power, Hydrophobic, Silane, Hydration process, Mechanical performance

## Abstract

To address the potential adverse impact of silane on cement hydration, this study systematically investigates the effects of power ultrasound on the mechanical and hydrophobic properties, and the microstructure of silane-modified cement paste. Experimental results showed that ultrasonic treatment within the 240–720 W range significantly enhanced the compressive strength compared to the reference group. In particular, the 480 W group exhibited increases in compressive strength of 36.9 %, 10.7 %, and 7.9 % at 1, 3, and 28 days, respectively. X-ray diffraction and TG analysis revealed that this treatment accelerated the dissolution and hydration degree of the clinker. Contact angle measurements indicated that optimal hydrophobic performance was achieved at 720 W, with a contact angle of 133.0°, representing a 34.2 % increase compared to the reference group. These findings indicate that power ultrasound holds excellent potential for preparing the hydrophobic cementitious materials.

## 1 Introduction

The water resistance of cement-based materials remains a pivotal challenge in modern construction, leading to yearly high maintenance costs. Water ingress through capillary action is the primary degradation mechanism [1]. Hydrophobic treatment is a crucial strategy for enhancing the water resistance of cement-based materials [2]. By forming a hydrophobic layer on the surface or within the pores via agents such as silanes emulsion [3], silicone resins [4], and silane coupling agents [5], water absorption and penetration are significantly reduced. This processing is particularly beneficial in frosty regions, marine environments, and the preservation of historical buildings.

Silanes have been the most widely used hydrophobic materials since their first synthesis by Friedel and Crafts in 1863 [6]. The typical structure of silanes is (RO)_3_-Si-X, where −OR (alkoxy group) is the key reactive group that facilitates the bonding of silanes. In the alkaline environment of cement paste, the alkoxy group reacts with water to generate hydroxyl groups (–OH). The resulting silanol ((HO)_3_-Si-X) undergoes dehydration condensation with hydroxyl groups (–OH) present in the hydration products such as C-S-H gel and portlandite (CH), forming covalent bonds such as Si-O-Si or Si-O-Ca [7]. This process forms a dense hydrophobic siloxane network on the surface and within the pores of cement-based materials [8], as described by **Eqs.** (1), (2). The −X groups in silanes include methyl (–CH_3_), ethyl (−C_2_H_5_), and amino (–NH_2_) groups [9]. Although these-X groups do not directly participate in chemical bonding with hydration products, they contribute to the spatial arrangement on the material surface, forming a low surface energy hydrophobic layer that inhibits moisture penetration and aids in constructing a water-repellent surface [10]. Feng et al. [11] demonstrated that incorporating a silane emulsion into concrete can significantly reduce the water absorption rate of modified concrete by more than 80 %, greatly enhancing the chloride ion corrosion resistance of embedded steel bars. Mora et al. [12] reported that modifying mortar with hydrophobic silica particles derived from dodecyl (−C_12_H_25_) resulted in strong hydrophobicity, reducing total water absorption by 45 % compared to the referenced group. A.M. Barberena-Fernandez et al. [12] investigated cement paste’s chemical and physical properties after adding TEOS (tetraethoxysilane). They reported that the interaction between TEOS and hydration products extended the chain length of the C-S-H gel and improved the bonding capacity of the matrix. Moreover, combining silanes with nanomaterials can further enhance the performance of the modified cement matrix. Zhang et al. [13] reported that emulsions were successfully created using graphene oxide and isobutyl triethoxysilane, as both substances can undergo covalent bonding. This bonding process generated sites for isobutyltriethoxysilane, which contributed to the improved water resistance of the concrete. Geng Y et al. [14] synthesized a silane-functionalized graphene oxide coating, significantly reducing cumulative water uptake and sorptivity.

1. Hydrolysis of Silane:

(1) $$({\text{RO)}}_{3}{\;\text{- Si - X + 3H}}_{2}\text{O}\to ({\text{HO)}}_{3}\;\text{- Si - X + 3ROH}$$

2. Dehydration Condensation with Cement Hydration Products:

(2) $$({\text{RO)}}_{3}\;\text{- Si - X + C - S - H or CH}\to {\text{Si - O - Si or Si - Ca bonds + 3H}}_{2}\text{O}$$

While silanes have demonstrated a substantial ability to improve the water resistance of cement-based materials, several technical challenges and limitations persist in their practical application: (i) dispersion and compatibility challenges in cement paste. When silane is directly mixed with cement paste, this can lead to localized accumulation and non-uniform coverage, potentially resulting in incomplete or slow hydrolysis/condensation of the silane and impeding the development of a continuous Si–O–Si (siloxane) network [15]; (ii) silanes may have potential limiting effects on cement hydration. Chen et al. [16] reported that silanes act as retardants in cement paste during the initial stages of hydration. The intermediate hydrolysis products between silanes and the hydration products can create a gel-like barrier that envelops the cement particles, delaying the hydration process. Kong et al. [17] demonstrated that both APTES (3-aminopropyltriethoxysilane) and AEAPTMS (N-2-aminoethyl-3-aminopropyltrimethoxysilane) significantly prolonged the induction period and reduced the degree of hydration. Similarly, GPTES (3-glycidoxypropyltriethoxysilane) and MAPTMS (methacryloxypropyltrimethoxysilane) were confirmed to retard the cement hydration during the hydrolysis and polycondensation processes, decreasing the compressive strength [11]; (iii) penetration into delicate capillary pores may be limited, reducing in-depth hydrophobization. To effectively establish a hydrophobic layer, silanes must penetrate the internal pore structure of cement-based materials. However, the extent of this penetration is markedly affected by the inherent characteristics of the surface [18].

Power ultrasound is a promising technology for addressing silane issues, characterized by ultrasonic waves operating within a frequency range of 20–100 kHz [19,20]. Within cementitious systems, ultrasound propagates as a mechanical wave, inducing oscillatory motion that enhances particle interactions. This wave propagation is intrinsically linked to ultrasonic cavitation, wherein the alternating pressure regions generated by the mechanical waves lead to microbubbles’ formation, growth, and implosive collapse. These cavitation events create localized extreme conditions (up to 5000 K and 1000 atm), generating shockwaves, microjets, and shear forces [21,22]. Such effects effectively disrupt the agglomeration of silane particles, facilitating their uniform dispersion within the medium and promoting hydrolysis reactions that generate additional silanol groups [23]. Moreover, power ultrasound can expedite mineral phase formation processes, enhancing the nucleation of calcium silicate hydrate (C-S-H) gel and calcium hydroxide (CH), thereby accelerating the hydration process [24]. This helps mitigate the potential adverse effects of silanes on cement hydration. Moreover, the abundant C-S-H gel and CH generated as hydration products possess hydroxyl groups, which may promote condensation reactions with silanes, thereby facilitating the formation of a dense hydrophobic network structure. Although some hypotheses were proposed, the synergistic effects of power ultrasound and silane have yet to be substantiated through experiments. Previous studies have predominantly focused on applying ultrasound in pure cement systems or nanoparticle dispersions [25,26], with limited attention to organic–inorganic hybrid systems such as silane-modified cement composites. However, the effects and mechanisms of ultrasonic power on the mechanical and hydrophobic properties of silane-modified cement-based materials remain unclear, especially in organic–inorganic composite systems.

Therefore, the objective of this study is to clarify the effects of ultrasound on silane dispersion, cement hydration, and the formation of hydrophobic networks, and to optimize ultrasonic parameters to enhance overall material performance. This study provides a mechanistic and quantitative framework for coupling silane modification with power ultrasound in an organic–inorganic cementitious system. Unlike prior work focusing on ultrasound in neat cement or inorganic nanoparticle systems, we systematically link ultrasonic power to silane dispersion, hydrolysis/condensation kinetics, and hydration evolution. The hydrophobic performance of the matrix was studied using the capillary water absorption test and the contact angle test. The microscopic composition and morphology were examined using thermogravimetric analysis (TGA), X-ray diffraction (XRD), Fourier transform infrared (FTIR), and scanning electron microscopy (SEM). Additionally, the dispersion of silane-modified hydrophobic cement pastes was also explored. The results indicate that moderate ultrasonic power promotes the early hydration reactions of cement minerals and enhances the dispersibility of silane, thereby improving the cement matrix’s compressive strength and hydrophobic properties. However, when the ultrasonic power reached 960 W, the excessive acoustic energy may significantly increase the water absorption rate and decrease the contact angle. These findings provide practical guidance for tuning ultrasonic parameters to achieve robust and durable water resistance, offering a solid theoretical basis and technical support for the efficient fabrication of water-resistant cement-based materials.

## 2 Raw materials and methods

### 2.1 Raw materials

**Fig. 1:**
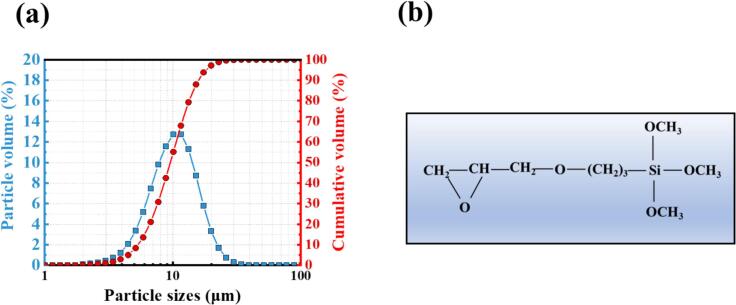
(a) The particle size distribution of HSRPC, (b) Chemical structure of KH560.

The cement used in this study is a P·I 42.5 type cement from Fushun Cement Co., Ltd. Table 1 presents the main oxides and mineral phases. The particle size distribution of the cement is shown in Fig. 1(a). The silane emulsion used in this experiment was designated SILRES® BS 1808 CN and provided by Wacker Chemie (China) Co., Ltd. SILRES®. The basic physical and chemical properties of the BS1808 CN are presented in Table 2. Its main chemical formula is γ-(2,3-epoxypropoxy) propytrimethoxysilane (KH560), as shown in Fig. 1(b).

**Table 1:** The composition of the main oxides and mineral phases of P.I 42.5.

XRF		XRD-Rietveld	
Main oxides	wt.%	Phases	wt.%
CaO	66.76	C_3_S	63.4
SiO_2_	18.08	C_2_S	12.9
Al_2_O_3_	4.18	C_3_A (cubic + ortho)	6.5
Fe_2_O_3_	3.76	C_4_AF	7.4
SO_3_	1.88	Gypsum	2.0
Na_2_O	0.24	Bassanite	0.3
K_2_O	0.60	Anhydrite	n.s.
MgO	1.88	Calcite	2.0
TiO_2_	0.34		
LOI	1.88		

n.s.: phase content below the reliability limit.

**Table 2:** The physical and chemical properties of SILRES® BS 1808 CN.

Appearance	Solid Content (%)	Boiling Point (°C)	Density (g/cm^3^)	Viscosity (mPa·s)
Milky white liquid	40	100	0.95	12

### 2.2 Preparation methods

**Fig. 2:**
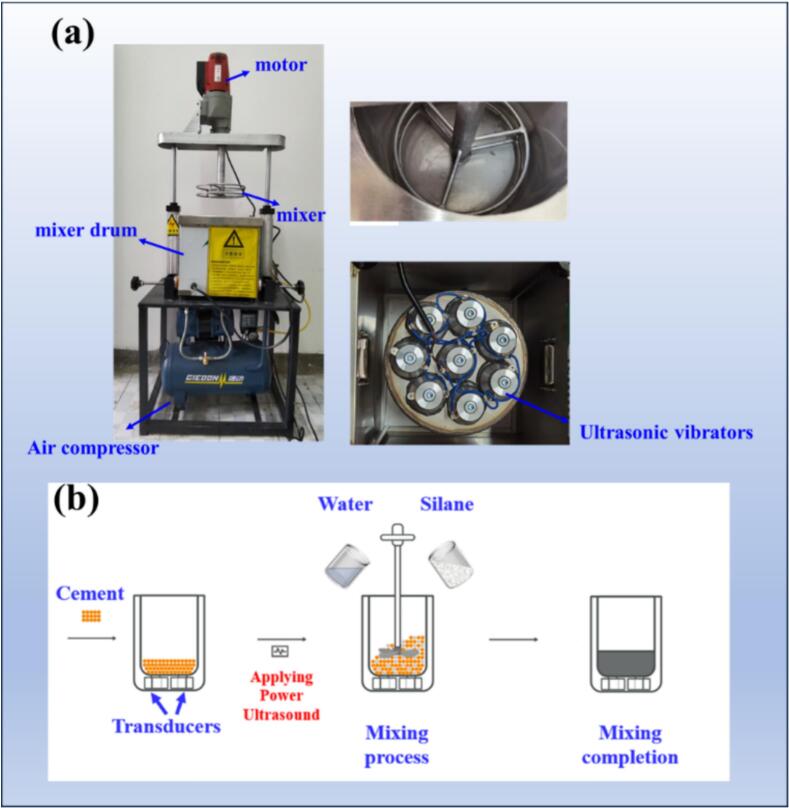
Ultrasound equipment used for the test.

The ultrasonic equipment used in this experiment included a power ultrasonic generator and an ultrasonic reactor equipped with piezoelectric transducers for assisted mixing. The appearance of the ultrasonic equipment is shown in Fig. 2. The main body of the ultrasonic tank is a cube with a side length of 281 mm, which is embedded with a cylindrical component measuring 240 mm in diameter and 197 mm in height. The bottom of the tank is equipped with eight ultrasonic transducers connected in series and parallel configurations. Each ultrasonic transducer operates at a frequency of 28 kHz, with an output power ranging from 110 W to 120 W, allowing for a total adjustable ultrasonic power of 0–960 W. The mechanical mixing device used in this experiment is a helical mechanical stirrer produced by Riejies Company, with a rated power of 2800 W. An air compressor is used to control the rise and fall of the electrical mixer. The temperature of the mixing water was maintained at 25 °C. Silane-modified hydrophobic cement pastes with a water-to-binder (w/b) ratio of 0.40 were prepared. When the silane emulsion replaced 1 % of the cement’s mass, five different ultrasonic power levels were established: 0 W, 240 W, 480 W, 720 W, and 960 W, corresponding to ultrasonic intensities of 0 W/cm^2^, 0.53 W/cm^2^, 1.06 W/cm^2^, 1.59 W/cm^2^, and 2.12 W/cm^2^, respectively. The mixture proportions are presented in Table 3.

**Table 3:** Mixture proportions for different ultrasonic powers.

w/b ratio	Silane Dosage (%)	Mixing time (min)	Power (W)	Cement (g)	Water (g)
0.40	1	3	0	2000	788
0.40	1	3	240	2000	788
0.40	1	3	480	2000	788
0.40	1	3	720	2000	788
0.40	1	3	960	2000	788

First, the cement was poured into the ultrasonic tank, followed by the addition of water and silane emulsion. The corresponding ultrasonic power was activated and the mixing process commenced. Mechanical mixing was conducted simultaneously at a speed of 150 rpm for 3 min to ensure thorough mixing. After mixing, the mixture was poured into a designated mold measuring 40 mm × 40 mm × 40 mm, compacted through vibration, and covered with a layer of cling film to reduce moisture evaporation. All the samples were subjected to external ambient room temperature curing for 1 day. Following the removal of the molds, the samples were placed in a standard curing room (humidity > 95 %, temperature 20 ± 2 °C) for curing until the corresponding testing age was reached.

### 2.3 Characterization methods

#### 2.3.1 Compressive strength test

After the samples were cured to the corresponding testing age, they were removed from the standard curing room. The surfaces of the samples were wiped with a damp cloth until they reached a saturated surface-dry condition. Then they were placed in a cement mortar compressive strength testing machine. The loading rate was set at 2.4 kN/s, and the samples were subjected to loading until failure, with the applied load recorded at the moment of rupture. The strength was determined as the average value of three specimens. The entire process was conducted in accordance with ASTM C109 [27].

#### 2.3.2 Capillary water absorption test

The capillary water absorption test followed the traditional weighing method of ASTM C1585-13 [28]. After curing for 28 days, the samples were removed from the standard curing room and placed in an oven at 50 ± 2 °C for 3 days. To facilitate unidirectional moisture transfer, the bottom surfaces were submerged in water, whereas the remaining sides were hermetically sealed with paraffin wax to prevent lateral moisture ingress. Additionally, cling film was used to cover the bottom surface, which was secured with a small rubber band to prevent moisture loss and evaporation.

**Fig. 3:**
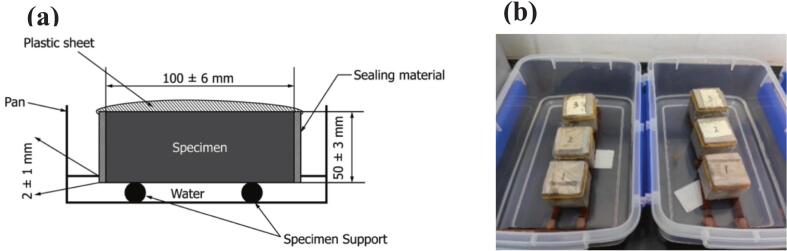
Capillary water absorption test: (a) schematic diagram of the capillary water absorption test [28] and (b) capillary water absorption test.

A custom capillary water absorption device consisting of a bottom tray and support rods was fabricated following ASTM C1585-13 standards. A schematic diagram of the device and the actual experimental setup is depicted in Fig. 3. During testing, the forming surface of the sample was placed downwards into the device to make contact with water, and timing commenced when the sample surface formed contact with the water. The mass of each sample was measured at the time intervals indicated in Table 4, and the water absorption mass for each set of three samples was recorded to ensure reproducibility of the experiment.

**Table 4:** Time interval of the capillary water absorption test.

Time	60 s	5 min	10 min	20 min	30 min	60 min	2–6 h Once an hour	1–8 days, Once a day
Permissible error	2 s	10 s	2 min	2 min	2 min	2 min	5 min	2 h

#### 2.3.3 Contact angle test

The contact angle is an essential parameter for characterizing the hydrophilicity or hydrophobicity of materials. The capillary water absorption process simulates water ingress in materials under natural wetting conditions, providing critical insights into the liquid transport capacity of their internal pore networks [29]. In silane-modified hydrophobic cement paste, the emulsion mitigates capillary forces by reducing the surface energy at the pore-solid interface and physically obstructing capillary channels [30]. After curing for 28 days, the samples were removed from the curing chamber. Initially, the samples were sliced via a QG-4A-T dual-hand-operated metallographic cutting machine produced by Shaoxing Jingbo Testing Instruments Co., Ltd., which was cut in half to create a flat surface. The surfaces of the specimens were polished using 2000-grit abrasive paper and subsequently rinsed with water for cleaning. Once the surfaces of the test samples had naturally air-dried, the static and dynamic contact angles of the surfaces from different ultrasonic power groups with water droplets were measured using a Kruss DSA100 contact angle analyzer from Germany. A droplet volume of 5.0 ± 0.1 µL was dispensed at a dosing rate of 0.5 µL/s through a 30G stainless-steel needle. Measurements were performed at 23 ± 2°C and 50 ± 5 % relative humidity. Before testing, specimens were cured as described in Section 2.2 and then equilibrated in the testing environment for 24 h; any loose surface dust was removed using oil-free air. Images were recorded immediately after deposition (t = 0 s) and subsequently at 30 s intervals up to 120 s. For each group, five droplets were measured on each of three replicate specimens.

#### 2.3.4 Microscopic composition test

The samples were crushed into small pieces and soaked in anhydrous ethanol to terminate the hydration process at the designated age. The anhydrous ethanol was replaced after soaking for 1 day and again after 3 days. Following the final soaking, the samples were placed in a vacuum drying oven at 40 °C to dry. After drying, the samples were manually ground via a mortar and pestle and passed through a 75 μm sieve for sealed storage until analysis. The hydration products of the cement paste were characterized utilizing a PANalytical X’Pert powder diffractometer from Spectris Pte. Ltd., which is equipped with Cu-Kα radiation (λ = 1.54 Å). The powdered samples were subjected to X-ray diffraction analysis over a 2θ range of 5°–70°, employing a step size of 0.02° at an operating voltage of 35 kV and a current of 40 mA. This study used analytically pure CaF2 (≥99.7 wt%) as the external standard to investigate the effect of PUS-assisted mixing on the mineral phases of silane-modified cement paste. Quantitative X-ray diffraction (XRD) pattern analysis was adopted using the Rietveld refinement technique. The crystalline phases were quantified with Highscore Plus 5.2 software, referencing the Crystallography Open Database (COD).

The sample preparation method for TGA and FTIR was the same as for XRD. Thermogravimetric analysis (TGA) was performed via a TGA/DSC 1/1600 SF synchronous thermal analyser produced by Mettler Toledo, with a testing temperature range from room temperature to 900 °C and a heating rate of 10 °C/min in a nitrogen atmosphere. Fourier transform infrared spectroscopy (FTIR) was conducted via a Nicolet iS50 spectrometer produced by Thermo Fisher Scientific, with a resolution of 4 cm^−1^, a wavenumber accuracy of 0.005 cm^−1^, and a spectral range of 400–4000 cm^−1^.

#### 2.3.5 Morphology test

Sample micromorphology was detected using scanning electron microscopy (SEM). The sample preparation method involved crushing and selecting slices with a diameter of approximately 8 mm and a 3–5 mm height. The hydration process was terminated using anhydrous ethanol, following the same procedure used for the XRD samples. The samples were then placed in a vacuum drying oven at 40 °C to dry.

Subsequently, the samples were securely attached via conductive adhesive and subjected to gold sputtering treatment (20 s, repeated 5 times). They were kept in a vacuum environment until formal testing. The microscopic morphology was observed via a VEGA 3 LMH tungsten filament scanning electron microscope produced by TESCAN. The operating voltage of the electron microscope was set at 15 kV, with an electron beam intensity of 8.0, and the working distance was maintained between 8 and 15 mm.

#### 2.3.6 Zeta potential

The mutual repulsion induced by surface charges on particles prevents aggregation, enabling stable dispersion within the system. The zeta potential is a direct indicator of the repulsive forces between colloidal particles and can be employed to characterize the stability of colloidal systems. In this experiment, the prepared cement paste was transferred into centrifuge tubes and subjected to centrifugation at 3000 rpm for 5 min. Following centrifugation, the supernatant was carefully collected and retained for subsequent testing. Zeta potential measurements were conducted using a Zetasizer Nano ZS90 laser particle size analyser (Malvern Instruments Ltd., UK), with strict adherence to standardized protocols for assessing the colloidal stability of cement-based materials. This methodology ensures the elimination of sediment interference while preserving the intrinsic interfacial electrical properties of the colloidal system. The measurements were performed at a controlled temperature of 25 ± 1 °C. The pH of the suspensions was maintained within the range of 12.5 ± 0.2, corresponding to the highly alkaline nature of cement pore solution. Each sample was measured five times, and the average value was reported to ensure reproducibility.

## 3 Results and discussion

### 3.1 Compressive strength

**Fig. 4:**
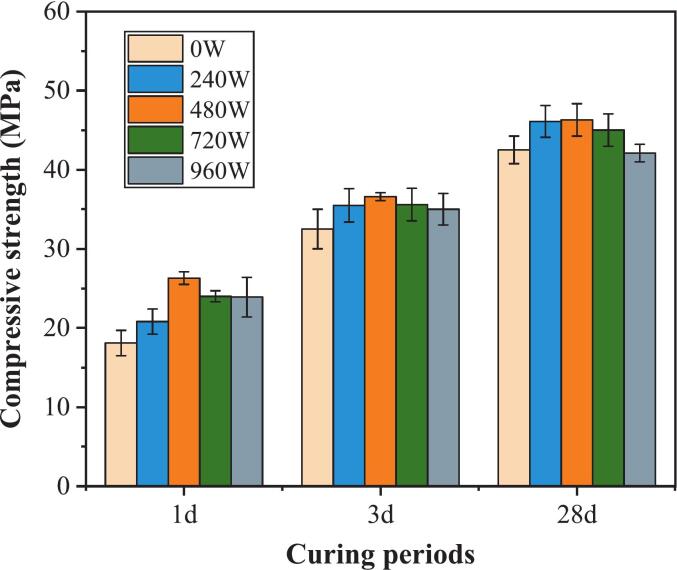
Effect of ultrasonic power on the compressive strength of cement paste.

Fig. 4 shows the evolution of the compressive strength of the silane-modified hydrophobic cement paste under different ultrasonic powers after 1 day, 3 days, and 28 days of curing. The data trend indicated a nonlinear response characteristic, where the compressive strength of the samples initially increased significantly with increasing ultrasonic power (240 W and 480 W), followed by a gradual decline at higher power levels (720 W and 960 W). The samples achieved optimized compressive strengths of 23.6 MPa, 38.9 MPa, and 52.7 MPa at 1, 3, and 28 days, respectively, under 480 W, representing increases of 36.9 %, 10.7 %, and 7.9 % compared to the reference group. However, the compressive strength declines when the ultrasonic power exceeds 480 W. The 960 W group decreased by approximately 8–12 % compared to the 480 W group. In fact, our previous research also included experiments using the same cement type with a pure cement paste group (without silane) under various ultrasonic power conditions [25]. The compressive strengths of batches applying 480 W and 912 W increased by 5.0 % and 26.1 %, respectively, at 1 day, and increased by 5.5 % and 18.3 %, respectively, at 28 days compared with the reference group without ultrasound. When comparing these results with those of the silane-modified groups, it is evident that the combination of silane modification and ultrasonic treatment yields a more pronounced enhancement in mechanical strength. This direct comparison demonstrates the synergistic effect of silane and ultrasonic treatment, thereby substantiating the advantages of our proposed approach.

### 3.2 Hydrophobic performance

#### 3.2.1 Capillary water absorption

**Fig. 5:**
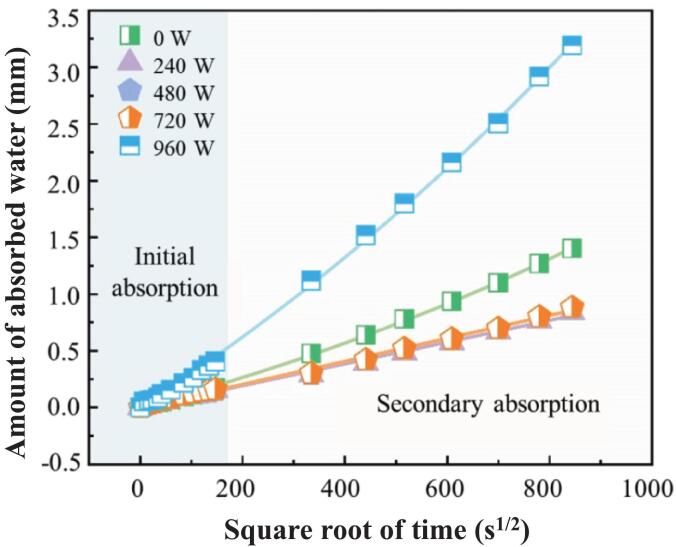
Effect of ultrasonic power on capillary water absorption.

Fig. 5 shows the relationship between cumulative water absorption and the square root of time (S^1/2^), demonstrating distinct patterns across the ultrasonic groups. The cumulative water absorption process under ultrasonic power treatment exhibited a quick growth trend with S^1/2^, which can be categorized into two distinct stages: an initial rapid growth stage (S^1/2^ = 0–147  s) and a subsequent slow-growth stage (S^1/2^ = 147–844 s). During the initial phase, rapid water ingress occurred due to capillary forces immediately after the bottom surface of the sample contacted water, leading to swift pore filling. The absorption rate then decreased and stabilized in the later phase. Compared with the referenced group (0 W), the samples treated with ultrasonic power levels of 240 W, 480 W, and 720 W presented significantly lower cumulative water absorption. However, the high-power group (960 W) displayed unexpected behaviour, surpassing the referenced group’s cumulative water absorption and absorption rates. Subsequent TGA results indicated that 960 W ultrasonic treatment reduced the chemically bound water content within the samples. This reduction suggests that the hydration process was adversely affected, leading to insufficient formation of hydration products such as C-S-H, which further exacerbates the susceptibility of the matrix to moisture penetration and increases capillary porosity.

The sample’s capillary water absorption was calculated according to ASTM C1585-13, as expressed in **Eq.** (3).

(3) $$I=\frac{m_{t}}{a\times d}$$

where *I* represents the capillary water absorption content (mm); *m_t_* represents the change in specimen mass at time *t* (g); *a* represents the contact area between the sample and water; and *d* represents the density of water.

As expressed by Eq. (4), the capillary water absorption *I* is linearly related to the square root of time.

(4) $$I=W_{S}\sqrt{t}$$

where *W_S_* is the water absorption rate, *t* is the elapsed time (min).

The results of the capillary water absorption tests were fitted in segments to obtain the samples’ initial and secondary water absorption rates. The fitting results are shown in Table 5.

**Table 5:** Fitting equations for water absorption at different stages for power groups.

Ultrasonic power	Fitting Equations			
	Initial water absorption rates	R^2^	Secondary water absorption rates	R^2^
0 W	y = 0.00104x	0.98	y = 0.00181x + 0.15014	0.99
240 W	y = 0.00096x	0.95	y = 0.00107x + 0.07657	0.99
480 W	y = 0.00095x	0.95	y = 0.00113x + 0.07918	0.99
720 W	y = 0.00106x	0.97	y = 0.00112x + 0.06894	0.99
960 W	y = 0.00263x	0.99	y = 0.00406x + 0.27529	0.99

**Fig. 6:**
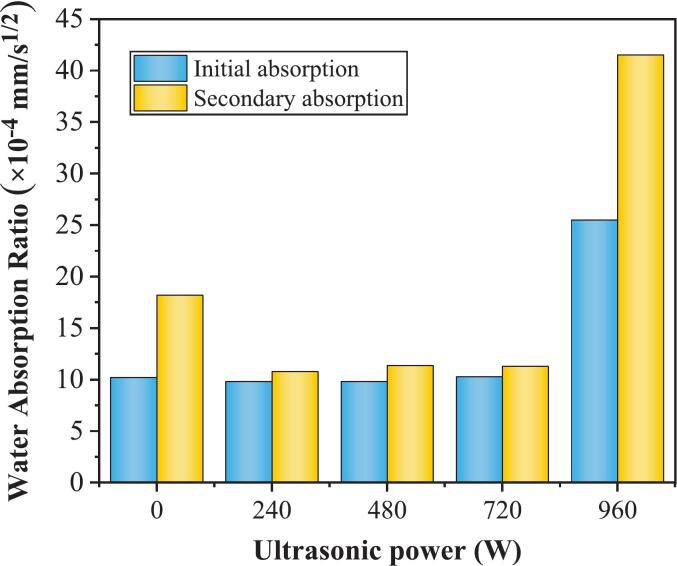
Effect of ultrasonic power on water absorption.

The initial and secondary water absorption rates of the samples, derived from the fitted equations, are plotted against ultrasonic power in Fig. 6. The results demonstrate that ultrasonic power significantly enhances the water absorption performance of the samples. As the ultrasonic power increased (0–960 W), the initial and secondary water absorption rates exhibited similar trends. Specifically, at ultrasonic powers of 240 W, 480 W, and 720 W, the initial water absorption rate initially decreased, followed by an increase. However, when the ultrasonic power was increased to 960 W, a significant escalation in the initial water absorption rate was observed, reaching as high as 2.5 times that of the reference group. Notably, the influence of ultrasonic power on the secondary water absorption rate is more pronounced. Compared with those of the referenced group (0 W), the secondary water absorption rates under 240 W, 480 W, and 720 W ultrasonic powers decreased by 40.8 %, 37.6 %, and 38.1 %, respectively. In contrast, at 960 W, the secondary water absorption rate increased twofold. These results suggest that there exists a critical threshold for enhancing the water resistance behavior of the silane-modified hydrophobic cement paste, which ranges from 720 W to 960 W, corresponding to ultrasonic intensities of 1.59 W/cm^2^ and 2.12 W/cm^2^, respectively.

The analysis indicates that power ultrasound within a specific range (240–720 W) effectively reduces the capillary water absorption rate of the samples. Moderate ultrasonic power promoted the formation of hydrophobic films through accelerated interactions between the silane emulsions and the cement matrix. These films effectively fill capillary pores, suppressing water ingress. Relative to the 240 W–720 W groups, the 960 W group exhibits markedly higher initial and secondary sorptivity. This deterioration arises from the combined effects of: (i) High ultrasonic power may induce silane agglomeration and inhibit the dehydration-condensation reaction of hydration products, as evidenced by the attenuation of the Si–O–Si band at ∼ 1130 cm^−1^ in FTIR spectra. This defective cross-linking compromises the structural integrity of the hydrophobic network, ultimately impairing resistance to water ingress; (ii) Excessive ultrasonic power suppresses the hydration process, evidenced by elevated residual C_3_S content at 28 days in QXRD analysis and previous studies [25]. This incomplete reaction yields insufficient hydration product formation, which coarsens the pore structure. Earlier studies also proved the pore structure of samples under various ultrasonic powers, revealing that high-intensity PUS, such as 912 W, may compromise the interlayer space during the later curing period, leading to a reduction in gel pores and an increase in the total number of capillary pores [31]. These findings delineate a non-monotonic ultrasonic power window in which moderate power (240–720 W) promotes emulsion dispersion, silane hydrolysis, and pore refinement, whereas excessive power (960 W) proves counterproductive.

#### 3.2.2 Contact angle test

**Fig. 7:**
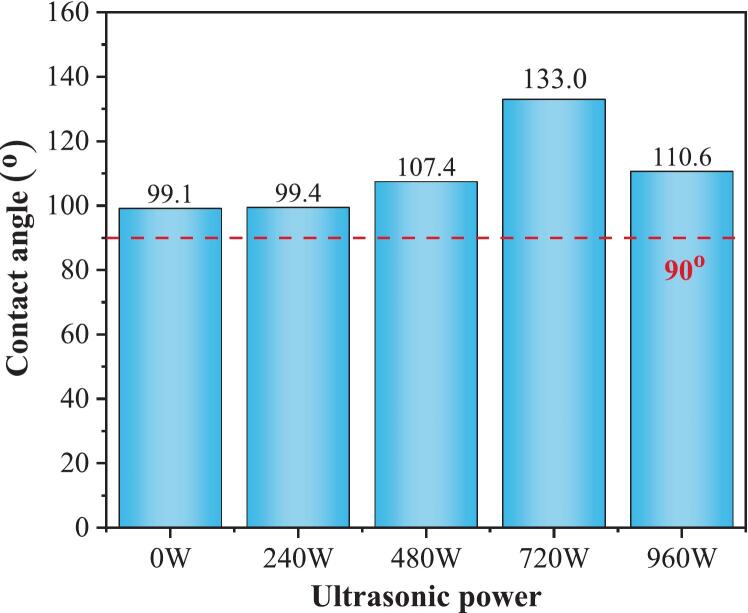
Contact angle at different ultrasonic powers.

The primary objective of this section was to systematically investigate the influence of variations in ultrasonic power on the hydrophobicity of silane-modified hydrophobic cement paste. The magnitude of the contact angle is an essential criterion for assessing the wettability of surfaces. A contact angle of less than 90° indicates hydrophilicity, whereas a contact angle greater than 90° signifies hydrophobicity [32]. The results of the contact angle tests at different power levels (0 W, 240 W, 480 W, 720 W, and 960 W) are presented in Fig. 7. The contact angles obtained at these power levels were 99.1°, 99.4°, 107.4°, 133.0°, and 110.6°, respectively. The 720 W group exhibited the maximum contact angle, representing a 34.2 % increase compared to the 0 W referenced group. The microvibrations induced by ultrasonication also aid in penetrating the silane solution into the capillary pores [37]. Meanwhile, it accelerates silane hydrolysis and condensation reactions, facilitating the formation of stable Si-O-Si bonds with hydroxyl groups, as proved by the FTIR test. However, the contact angle at 960 W decreased significantly compared to other ultrasonnic groups, indicating that excessive ultrasonic power may inhibit the dehydration condensation between silane and cement hydration products.

**Fig. 8:**
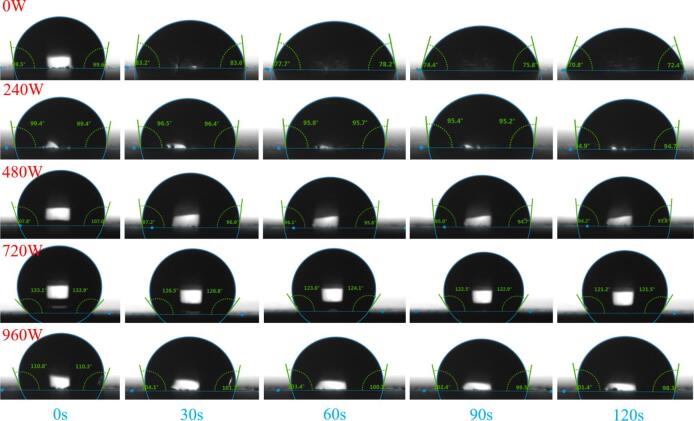
Variation in the contact angle with time for different ultrasonic power groups.

To observe the stability of the contact angle on the sample surfaces, measurements were taken and recorded every 30 s. Fig. 8 shows the variation in the contact angles over time for each power group within 120 s from the start of the timing. The specific results are shown in Table 6. At 0 W, during the 120 s dynamic contact angle test, the water droplet exhibited significant collapse, with the contact angle decreasing from 98° to 71° and a reduction of 27° within 120 s. This finding indicates that moisture rapidly penetrated the substrate through defects or inadequately covered pores in the silane layer, leading to a gradual loss of hydrophobicity on the surface. In contrast, under the influence of 240 W of power, the morphology of the water droplet remained relatively stable over the same duration, with the contact angle decreasing from 99° to 95°, a mere reduction of 4°. Similarly, under ultrasonic treatment at 480 W, 720 W, and 960 W, the contact angles decreased by 13°, 12°, and 10°, respectively, within 120 s. These observations suggest that the stability of the contact angle is enhanced following ultrasonic treatment within the same time frame. The more uniform and stable rough structure created by power ultrasonic treatment can delay the collapse of the contact angle due to gravitational or permeation effects. Additionally, with ultrasonic assistance, silane can more effectively fill the pores and microcracks [25], reducing the number of localized deficiencies in the surface layer and enhancing the overall stability. When 960 W ultrasound was applied, the initial contact angle at 0 s was 110.55°, which slightly exceeded that of the 0–480 W group. This is inconsistent with the results of water sorptivity in Section 3.2.1. The static contact angle probes the outermost surface chemistry and topography. In contrast, the sorptivity of silane-modified cement paste is mainly governed by the pore structure and the characteristics of hydrophobic layers. Specifically, excessive power (960 W) inhibits silane crosslinking and yields patchy coverage (attenuated Si–O–Si at ∼ 1130 cm^−1^ in FTIR test in this study), producing hydrophilic defects and percolating pathways. Despite this, the 960 W group exhibits a high content of methyl groups (–CH_3_) compared to other groups, which is a potential hydrophobic group with low surface energy and low hydrophilicity, making it difficult for water molecules to approach and wet the surface [10]. Therefore, the initial contact angle of 960 W is higher than that of 0–480 W. The QXRD results indicate that higher residual C_3_S and lower chemically bound water suggest that high-power ultrasonication inhibits hydration, which also leads to decreased stability at higher power levels (960 W).

**Table 6:** Change in contact angle under different ultrasonic power groups.

		0 s	30 s	60 s	90 s	120 s
0 W	Left	98.5^o^	83.2^o^	77.7^o^	74.4^o^	70.8^o^
	Right	99.6^o^	83.6^o^	78.2^o^	75.8^o^	72.4^o^
	Average	99.05^o^	83.4^o^	77.95^o^	75.1^o^	71.6^o^
240 W	Left	99.4^o^	96.5^o^	95.8^o^	95.4^o^	94.9^o^
	Right	99.4^o^	96.4^o^	95.7^o^	95.2^o^	94.7^o^
	Average	99.4^o^	96.45^o^	95.75^o^	95.3^o^	94.8^o^
480 W	Left	107.8^o^	97.2^o^	96.1^o^	95.0^o^	94.2^o^
	Right	107.0^o^	96.6^o^	95.6^o^	94.7^o^	93.8^o^
	Average	107.4^o^	96.9^o^	95.85^o^	94.85^o^	94.0^o^
720 W	Left	133.1^o^	126.5^o^	123.6^o^	122.5^o^	121.2^o^
	Right	132.9^o^	126.8^o^	124.1^o^	122.9^o^	121.5^o^
	Average	133.0^o^	126.65^o^	123.85^o^	122.7^o^	121.35^o^
920 W	Left	110.8^o^	104.1^o^	103.4^o^	102.4^o^	101.4^o^
	Right	110.3^o^	101.7^o^	100.1^o^	99.5^o^	98.3^o^
	Average	110.55^o^	102.9^o^	101.75^o^	100.95^o^	99.85^o^

### 3.3 Microstructural evolution

#### 3.3.1 QXRD analysis

**Fig. 9:**
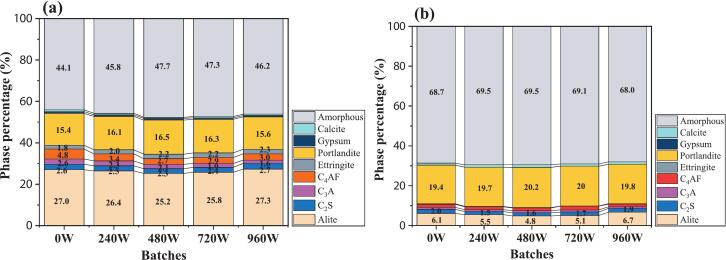
XRD patterns of the samples at different ultrasonic powers.

Fig. 9(a) indicates that ultrasonic power did not alter the hydration reaction pathway of silane-modified hydrophobic cement paste but influenced the quantity of products. At the 1 day hydration age, moderate power ultrasound (240–720 W) promoted hydration: content of C_3_S decreased by 2.22 %, 6.30 %, and 4.45 % compared to the 0 W group. In contrast, high power (960 W) exhibited an inhibitory effect. The intensities of the C_3_S diffraction peaks increased to levels comparable to the 0 W group. A similar trend was also observed at 28 days. Fig. 9(b) shows that when ultrasonic powers of 240 W, 480 W, and 720 W were applied, the C_3_S content decreased by 10.13 %, 21.57 %, and 16.67 %, respectively, compared to the 0 W condition. However, at 960 W, the C_3_S content increased by 9.48 % relative to 0 W. This may be due to the destructive interference of ultrasonic waves propagating through the viscous medium, adversely affecting the hydration. Thus, the amorphous content of C-S-H gel in the 960 W group was reduced by 0.94 %, 2.18 %, 2.17 %, and 1.54 % compared to the other ultrasonic groups.

#### 3.3.2 TG analysis

**Fig. 10:**
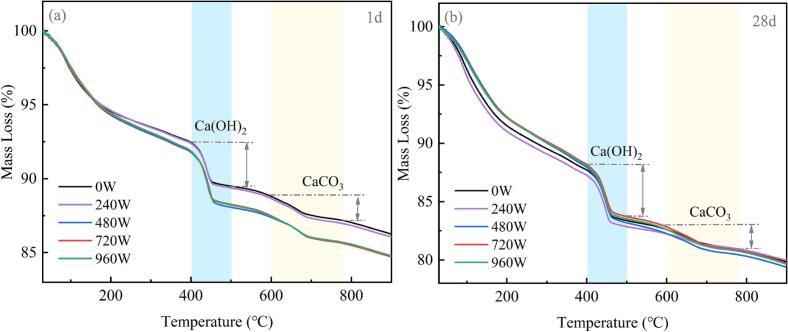
TG curves of the samples at different ultrasonic powers.

The thermogravimetric (TG) analysis presented in Fig. 10. The TG curve at 1 day for the 240 W group closely resembled that of the referenced group (0 W), exhibiting minimal mass loss within the 105–400 °C range, which implies relatively low formation of hydration products. The groups subjected to 480 W, 720 W, and 960 W presented increased mass loss compared with the referenced group, indicating that the ultrasonic energy facilitates the accelerated formation of hydration products.

The TG curve can be further analysed semiquantitatively to identify four primary weight loss stages associated with the hydration reaction: (1) From room temperature to 300 °C, dehydration occurs in several hydration products, including C-S-H gel, ettringite, monosulfate (AFm-Ms), and monocarboaluminate (AFm-CO_3_) [33]; (2) between 400 °C and 500 °C, dehydroxylation of CH takes place [34]; (3) from 600 °C to 780 °C, decarbonation of calcium carbonate occurs [35]; and (4) between 800 °C and 900 °C, decomposition of tobermorite, xonotlite, or wollastonite occurs [36].

Based on the aforementioned temperature ranges, the CH content can comprise the existing CH and the calcium carbonate formed through carbonation, as expressed in **Eq.** (5) [37,38]. Chemically bound water refers to water molecules that are chemically combined with cement mineral phases (such as C_3_S, C_2_S, C_3_A, and C_4_AF) to form hydration products (such as C-S-H gel, ettringite, and CH). Its content directly reflects the amount of water that has participated in the hydration reaction within the cement paste and can be represented by **Eq.** (6) [31].

(5) $$W_{\mathrm{CH}}=L_{\mathrm{CH}}\frac{\text{74}}{\text{18}}+L_{\mathrm{CaC}O_{3}}\frac{74}{44}$$

where *W_CH_* is the total CH content (%), *and L_CH_* and *L_CaCO3_* are the relative mass losses due to the dehydration of CH and CaCO_3_, respectively. 74, 44, and 18 are the relative molecular weights of CaCO3, CH, and H_2_O, respectively.

(6) $$W_{B}=L_{T}-L_{\mathrm{CaC}O_{3}}$$

where *W_B_* is the chemically bound water content (%), and *L_T_* is the total mass loss within the temperature range of 105–1000 °C.

**Fig. 11:**
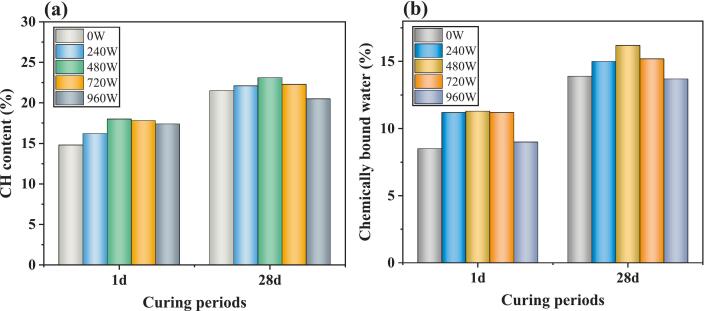
CH content and chemically bound water content of samples at different ultrasonic powers.

Fig. 11(a) shows that all ultrasonic groups (240–960 W) show higher CH contents than the referenced group. At 1 day, the 480 W group achieves a CH content of 17 %, a 3.26 % increase over the referenced group. In contrast, excessive ultrasonic power (960 W) may diminish the CH content. Fig. 11(b) presents the quantitative results of chemically bound water in cement paste under different ultrasonic power levels. Compared with the 0 W referenced group, the chemically bound water content at 240 W and 480 W significantly increased at both 1 day and 28 days of hydration. These changes are primarily due to power ultrasound’s thermal and cavitation effects, which facilitated the dissolution of C_3_S [39]. However, at 720 W and 960 W, the content of chemically bound water after 28 days of curing decreased by 6.1 % and 15.4 %, respectively, compared with the content at 480 W. This reduction indicates that further increasing ultrasonic power is detrimental to the late hydration process. In conjunction with the QXRD results, it suggests that the residual C_3_S may not receive sufficient water for subsequent hydration reactions during the later stages of curing [31,40].

#### 3.3.3 Morphology

**Fig. 12:**
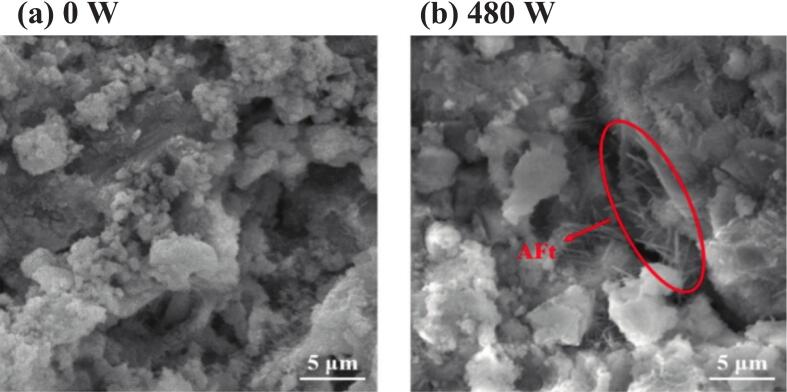
SEM images of samples at different ultrasonic powers.

Fig. 12(a) shows the morphology of the referenced samples, where the hydration products exhibit a clustered distribution with significant pore structure. In contrast, the sample treated with 480 W of ultrasonic power exhibits a relatively dense structure, as shown in Fig. 12(b). This improvement is due to the power ultrasound, which enables the nanometer-scale dispersion of silane molecules within the cement matrix [26]. It promotes the chemical bonding of siloxane chains with the hydration products, resulting in the formation of a continuous and dense hydrophobic membrane at the calcium‒silicate interface. Moreover, the needle-like morphology of ettringite crystals is well-formed, contributing to an interlocking reinforcement structure in three-dimensional space, thereby enhancing the strength [41].

#### 3.3.4 FTIR analysis

The analysis of the absorption characteristics of functional groups in silane-modified hydrophobic cement paste through Fourier transform infrared (FTIR) spectroscopy can elucidate the mechanisms by which ultrasonic treatment influences the chemical bonding of silane with cement hydration products, as well as the microstructure and hydrophobic properties. The characteristic peaks of the infrared spectra for the functional groups of the main substances in the silane-modified hydrophobic cement paste are presented in Table 7 [[42], [43], [44]].

**Table 7:** Characteristic absorption peaks of FTIR.

Wavenumber(cm^−1^)	Characteristic Peak Attribution
873.12	Out-of-Plane Bending Vibration
970.00	Stretching vibration of Si-O
1130.00	Stretching vibration of Si-O-Si
1425.83	Symmetrical stretching vibration of SO_4_^2−^.
2920.00	Asymmetric stretching vibration of –CH_3_
2970.00	Asymmetric stretching vibration of –CH_2_
3409.00	Stretching vibration of –OH

**Fig. 13:**
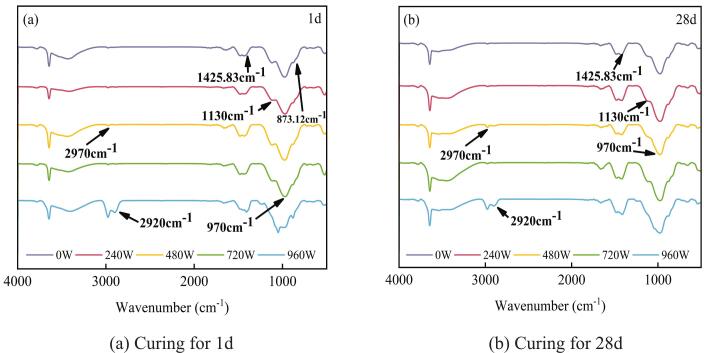
FTIR of different ultrasonic powers. (a) Curing for 1d. (b) Curing for 28d.

Fig. 13 shows the characteristic stretching vibration peaks of the Si-O bond at 970 cm^−1^ and the Si-O-Si bond at 1130 cm^−1^, which reflect the chemical reaction process between the silane and cement-based materials. According to the reaction mechanism of silane with cement-based materials, silane first undergoes hydrolysis under the influence of ultrasonic power to generate silanol (Si-OH). These Si-OH groups subsequently react with the hydroxyl groups (–OH) in the cement-based materials through dehydration condensation, forming Si-O-Si bonds. The areas of the –OH at 3409 cm^−1^ and Si-O-Si bond peaks at 1103 cm^−1^ in the 720 W ultrasonic group are significantly greater than those in the referenced group at 0 W, demonstrating that ultrasonic power can promote hydrolysis reactions.

In brief, hydrophobicity at the macroscale is governed not only by the abundance of low-surface-energy groups but also by the continuity and crosslinking degree of the siloxane network (Si–O–Si) and the underlying porosity effect. At 960 W, although the CH_3_/CH_2_ bands at 2920/2970 cm^−1^ become more pronounced, the Si–O–Si band near 1130 cm^−1^ is markedly diminished, indicating suppressed condensation and reduced crosslinking to the substrate. Moreover, high-power ultrasonication coarsens the pore structure and the stronger decay of the dynamic contact angle at 960 W. Thus, the mere presence of more organic moieties in the bulk does not translate into a higher apparent contact angle unless they are densely packed, properly oriented, and crosslinked at the outer surface and supported by a compacted microstructure.

Conversely, at 720 W, sufficient crosslinking (strong Si–O–Si), uniform coverage, and favorable micro-roughness promote a dense state, yielding the highest and most stable contact angles. Therefore, the non-monotonic trend arises from the interplay between surface chemistry (crosslinking/coverage and orientation) and pore structure, rather than from the CH_3_/CH_2_ content alone.

#### 3.3.5 Zeta potential

**Fig. 14:**
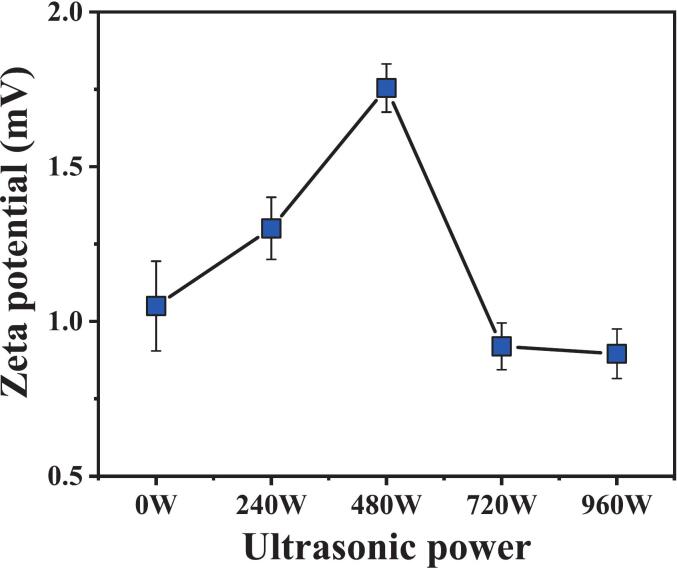
Effect of ultrasonic power on the zeta potential.

The zeta potential is a crucial parameter for assessing colloidal stability, as its absolute value directly correlates with the intensity of electrostatic repulsion between particles. Measuring the zeta potential offers significant insights into the surface charge characteristics, which is vital for comprehending suspensions’ stability and dispersion effectiveness [45]. The zeta potential of pure cement paste has been reported to be approximately − 11 mV or even lower, depending on the concentration of the cement suspension [46]. The variation in the zeta potential across different power levels for each experimental group is illustrated in Fig. 14. All the groups presented positive zeta potential values, all of which were below 2.0. For comparison, the zeta potentials of synthetic clinker phases in water are approximately − 5 mV for C_3_S, −7 mV for C_2_S, +12 mV for C_3_A, and + 5 mV for C_4_AF [47]. Moreover, the heterogeneous charge distribution across multiple phases provides selective adsorption sites for silane emulsion particles. Following the addition of the silane emulsion, due to the protonation of amino groups (–NH_3_^+^) in the organic silane under acidic conditions, some particles adsorb onto the surface of the cement particles through electrostatic interactions during the mixing process, resulting in a positively shifted zeta potential of the modified cement surface. In addition, the zeta potential values shown in Fig. 14 are relatively low. This is because the highly alkaline environment of the cement paste, rich in various ions such as Ca^2+^, Na^+^, and K^+^, can readily induce the double-layer compression effect [45]. This phenomenon may result in the aggregation or even destabilization of the emulsion or particles, thereby leading to a lower zeta potential [48].

The absolute value of the zeta potential of the silane-modified hydrophobic cement paste exhibited a significant nonlinear response to changes in ultrasonic power. As the ultrasonic power increases from 0 W to 480 W, the absolute value of the zeta potential increases significantly by 67.1 % from its initial value, reaching a maximum, indicating the formation of a stable particle system. However, as the power continues to increase to 720 W and 960 W, the absolute values decrease by 12.4 % and 14.7 %, respectively, compared with those of the referenced group, with the difference between the two values being less than 5 %. Notably, the zeta potential value at 960 W is slightly lower than that of the referenced sample. This non-monotonic variation highlights the intricate relationship between the colloidal dispersion system and ultrasonic power. At a moderate ultrasonic power of 480 W, the transient microjets and shock wave pressures generated by ultrasonic cavitation may deagglomerate secondary aggregates and promote the surface adsorption of silane molecules through mechanical shear, forming a stable steric hindrance layer. However, as the ultrasonic power is increased to 960 W, the overloaded acoustic field energy may lead to desorption and reorganization of the adsorbed silane molecules.

### 3.4 The action mechanism of different ultrasonic powers

**Fig. 15:**
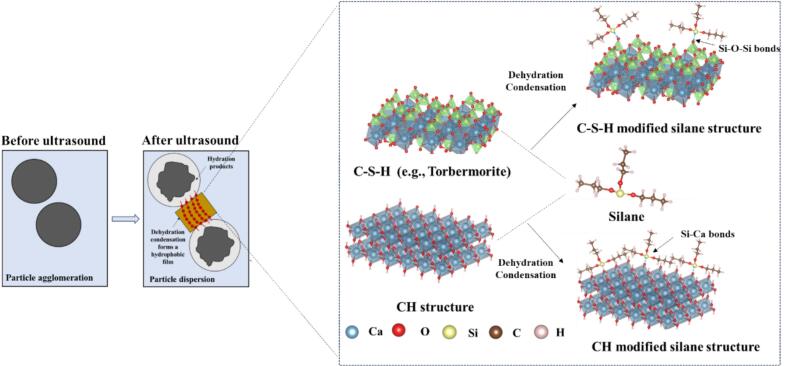
Mechanism of power ultrasound on silane-modified cement-based materials.

The role of the power ultrasound in these various stages is discussed, as depicted in Fig. 15**.** Moderate ultrasonic power (240–720 W) markedly improves the dispersion of silane emulsions within the cement matrix. The cavitation and mechanical vibrations generated by ultrasound disrupt the flocculated structure of cement particles, releasing free water, which results in a more homogeneous suspension. Moderate ultrasonic power accelerates the hydration of cement minerals, as evidenced by a reduction in C_3_S content and increases in CH and chemically bound water. Thus, hydrolysis and condensation reactions with CH or C-S-H gel under higher ultrasonic energy were accelerated, as proved by the FTIR test. These microstructural and chemical enhancements translate to superior macroscopic properties: the 480 W group achieves the highest compressive strength at all ages, with increases of 36.9 %, 10.7 %, and 7.9 % at 1, 3, and 28 days, respectively, compared to the reference. Furthermore, the contact angle reaches its maximum value of 133.0° at 720 W, representing a 34.2 % improvement over the reference.

When the ultrasonic power increased to 960 W, a pronounced detrimental effect on the microstructure and overall performance of silane-modified hydrophobic cement paste was observed. The TG analysis in Fig. 11 further revealed a decrease in chemically bound water content at 960 W, suggesting inhibited hydration reactions. Moreover, the capillary water absorption test (Fig. 5, Fig. 6) demonstrated a marked increase in both initial and secondary water absorption rates at 960 W, reflecting a coarsened pore structure and reduced water resistance. These findings are consistent with previous studies, which have reported that excessive ultrasonic energy can cause microstructural damage and disrupt the formation of hydration products in cementitious systems [25,31,49]. Such microstructural damage may facilitate rapid water ingress and undermine the integrity of the hydrophobic layer. Second, the high acoustic energy may induce destructive interference and chaotic molecular motion, which can hinder the hydrolysis and dehydration condensation reactions between silane and the hydration products of cement. This results in a reduction in the formation of Si-O-Si bonds, as evidenced by the FTIR analysis, and ultimately weakens the hydrophobic network within the matrix.

From an industrial perspective, while moderate ultrasonic power has been shown to enhance both mechanical strength and hydrophobicity, excessive ultrasonic energy can lead to adverse microstructural changes, reduce water resistance, and ultimately compromise the material's long-term durability. Furthermore, the design of ultrasonic transducer arrangements to precisely optimize and rigorously control the structural integrity and durability of hydrophobic cement-based products warrants further investigation.

## 4 Conclusion

1) The ultrasonic power of 240–720 W was highly effective in enhancing the compressive strength of the cement paste. In particular, the 480 W group exhibited increases in compressive strength of 36.9 %, 10.7 %, and 7.9 % at 1, 3, and 28 days, respectively, compared to the reference group. However, excessive ultrasonic power at 960 W severely hindered cement hydration and dehydration condensation with cement hydration products.
2) Compared with the nontreated group (0 W), the secondary water absorption rates under 240 W, 480 W, and 720 W ultrasonic powers decreased by 40.8 %, 37.6 %, and 38.1 %, respectively. The stability of the contact angle was optimal at 240 W, where it decreased by only 4° over 120 s. Notably, at an ultrasonic power of 720 W, the contact angle of the samples reached 133.0°, representing a 34.2 % improvement over that of the referenced samples. This enhancement was because power ultrasound accelerated hydrolysis and condensation, leading to a dense Si-O-Si network. However, excessively high power (960 W) can lead to cavitation energy overload, destroying the microstructure of the hydrophobic layer or causing disorder in the molecular arrangement, negatively impacting stability.
3) Ultrasonic power has a pronounced nonlinear effect on the dispersion of silane-modified cement paste. At a moderate ultrasonic power of 480 W, the absolute value of the zeta potential increases significantly by 67.1 % compared to its initial value, reaching a maximum and indicating the formation of a stable particle system. However, secondary particle aggregation occurs when the ultrasonic power is further increased to 960 W, resulting in a 14.7 % decrease in the zeta potential relative to the reference group.
4) Ultrasonic power does not modify the hydration reaction pathway of silane-modified cement paste but significantly influences the quantity of hydration products. Moderate ultrasonic power (240–720 W) promotes cement hydration, as evidenced by a marked reduction in C_3_S content. In contrast, high ultrasonic power (960 W) exerts an inhibitory effect on the hydration process. These findings underscore the necessity of optimizing ultrasonic parameters for enhanced performance.
5) These findings provide a scientific basis for designing high-efficiency ultrasonic protocols in cement modification, particularly for water-resistant or high-durability concrete applications. Further research should focus on multi-parameter optimization (e.g., frequency, duration) and in-situ characterization of ultrasonic effects on silane-cement interfaces. Additionally, the transition from laboratory-scale results to industrial processes remains a significant challenge for effective practical implementation.

## CRediT authorship contribution statement

**Guangqi Xiong:** Writing – original draft, Visualization, Methodology, Investigation, Formal analysis. **Ying Zhao:** Writing – review & editing, Resources, Methodology, Investigation. **Zheng Fang:** Writing – review & editing, Resources, Methodology, Investigation. **Yi Li:** Writing – review & editing, Formal analysis. **Yuanliang Ren:** Writing – review & editing. **Xiaolong Jia:** Writing – review & editing. **Bo Ran:** Writing – review & editing, Supervision. **Lei Xu:** Funding acquisition, Methodology, Supervision, Writing – review & editing. **Shuai Zhou:** Writing – review & editing, Supervision, Conceptualization. **Chong Wang:** Writing – review & editing, Supervision, Funding acquisition, Conceptualization.

## Declaration of competing interest

The authors declare that they have no known competing financial interests or personal relationships that could have appeared to influence the work reported in this paper.
